# Factors associated with concordance of mother-child (6–23 months) dietary diversity in Sub-Saharan Africa

**DOI:** 10.1371/journal.pone.0318493

**Published:** 2025-02-25

**Authors:** Meklit Melaku Bezie, Hiwot Altaye Asebe, Angwach Abrham Asnake, Bezawit Melak Fente, Zufan Alamrie Asmare, Alemayehu Kasu Gebrehana, Mamaru Melkam, Beminate Lemma Seifu, Sintayehu Simie Tsega, Yohannes Mekuria Negussie

**Affiliations:** 1 Department of Public Health, Institute of Public Health, College of Medicine and Health Sciences and Comprehensive Specialized Hospital, University of Gondar, Gondar, Ethiopia; 2 Department of Public Health, College of Medicine and Health Sciences, Samara University, Samara, Ethiopia; 3 Department of Epidemiology and Biostatistics, School of Public Health, College of Medicine and Health Sciences, Wolaita Sodo University, Sodo, Ethiopia; 4 Department of General Midwifery, School of Midwifery, College of Medicine & Health Sciences, University of Gondar, Gondar, Ethiopia; 5 Department of Ophthalmology, School of Medicine and Health Science, Debre Tabor University, Debre Tabor, Ethiopia; 6 Department of Midwifery, College of Health Sciences, Salale University, Salale, Ethiopia; 7 Department of Psychiatry, University of Gondar College of Medicine and Health Science, Gondar, Ethiopia; 8 Department of Medical Nursing, School of Nursing, College of Medicine & Health Sciences, University of Gondar, Gondar, Ethiopia; 9 Department of Medicine, Adama General Hospital and Medical College, Adama, Ethiopia; Mizan-Tepi University, ETHIOPIA

## Abstract

**Background:**

Inadequate dietary diversity is a major contributor to undernutrition, and compromises the health of both mothers and children. Available evidence demonstrates a significant association between maternal and child dietary diversity. However, there is limited evidence about the factors influencing the concordance of mother-child dietary diversity in Sub-Saharan Africa (SSA). Therefore, we investigated the factors associated with the concordance of mother-child dietary diversity in SSA.

**Methods:**

A community-based cross-sectional study was conducted based on the recent Demographic and Health Surveys (DHS) data of eight Sub-Saharan African countries. A total weighted sample of 29,138 mother-child pairs within the five years preceding the survey was included. A mixed-effect binary logistic regression model was employed to identify factors associated with concordance between mother-child dietary diversity. Variable with p-value < 0.2 in the bivariable mixed-effect binary logistic regression analysis was considered for the multivariable analysis. In the multivariable mixed-effect binary logistic regression analysis, the Adjusted Odds Ratios (AOR) with 95% Confidence Intervals (CI) were reported. The percentage of agreement between mothers and children with minimum dietary diversity was assessed using kappa statistics.

**Results:**

The concordance of dietary diversity between mother-child pairs in SSA was 74.48% (95% CI: 73.98, 74.98). A higher likelihood of mother-child dietary diversity concordance was significantly associated with mothers who had a primary level of education (AOR = 1.40, 95% CI: 1.31, 1.53) and those who were divorced or widowed (AOR = 1.54, 95% CI: 1.29, 1.84). Conversely, lower odds of concordance were observed among mothers with higher education (AOR = 0.75, 95% CI: 0.66, 0.85), those exposed to media (AOR = 0.78, 95% CI: 0.73, 0.83), and mothers belonging to poorer (AOR = 0.81, 95% CI: 0.74, 0.88), middle-income (AOR = 0.82, 95% CI: 0.75, 0.89), richer (AOR = 0.80, 95% CI: 0.73, 0.88), and richest (AOR = 0.75, 95% CI: 0.67, 0.83) households.

**Conclusion:**

The findings highlight that the dietary diversity concordance between mother-child pairs in sub-Saharan Africa is moderate at 74.48%. However, the factors influencing concordance suggest socio-economic and educational disparities. Mothers with a primary education level and those who were divorced or widowed had higher concordance with their children’s dietary diversity, indicating their potential prioritization of family dietary habits despite limited resources or support systems. On the other hand, lower concordance among mothers with higher education and those exposed to media suggest that these groups may adopt more individualized dietary practices. Furthermore, households with higher economic status, surprisingly, exhibited lower concordance, which may indicate resource allocation differences within wealthier families or greater dietary autonomy among children.

Globally, one-fourth of children aged 6–23 months and two-thirds of women of reproductive age suffer from micronutrient deficiencies, primarily due to inadequate dietary diversity [1–3]. It is estimated that 50–70% of the global disease burden is associated with poor dietary patterns and malnutrition [4]. In Low- and Middle-income Countries (LMICs), where many impoverished and rural households depend on starchy staples due to limited access to diverse foods, only 20% of children meet minimum acceptable dietary standards, exacerbating malnutrition and related health challenges [5,6]. Dietary Diversity (DD) is recognized as a key indicator of a healthy diet, reflecting the variety of food groups consumed within 24 hours [7]. According to the World Health Organization (WHO), DD serves as a proxy for child feeding practices, with consumption from at least four different food groups indicating that the child likely consumed an animal-source food, a fruit or vegetable, and a staple food such as grains, roots, or tubers [8].

## Background

Nutritional imbalances in early childhood are particularly critical, as children need energy- and nutrient-dense foods for healthy growth, alongside optimal breastfeeding [9–11]. Deficiencies during the first 1,000 days can lead to delayed cognitive development and alter the body’s ability to regulate energy and store fat, increasing the risk of chronic diseases later in life [12]. These nutritional shortfalls can also negatively impact academic performance and household economic stability, perpetuating the cycle of poverty [13,14]. In LMICs, about two-thirds of child deaths are linked to poor feeding practices, including inadequate dietary diversity [15,16]. Nutritional deficiencies, such as anemia, affect 58% of children under five, while approximately 200 million children globally experience stunting and wasting, with the problem being especially severe in African countries [17,18].

The WHO highlights the significant impact of maternal feeding practices on children’s dietary diversity, emphasizing the need for dietary alignment between mothers and their children [19]. Given that mothers play a crucial role in their children’s health, enhancing maternal nutrition is vital to breaking the cycle of intergenerational malnutrition [20]. Research identifies various factors affecting children’s dietary diversity, including maternal education, household wealth, location, media exposure, household size, parity, and access to postnatal care [21–24].

In LMICs, efforts to combat malnutrition have primarily focused on improving agricultural productivity, food quality, and micronutrient fortification programs [25,26]. However, there is increasing acknowledgment of the necessity to prioritize maternal nutrition in nutritional interventions. Extensive studies demonstrate that a mother’s diet significantly influences her child’s health and development by affecting breast milk quality, prenatal nutrition, and the child’s eating habits [27–29]. Therefore, promoting maternal nutrition is essential for enhancing maternal and child health outcomes, underscoring the need for targeted policies and programs focused on maternal diets.

Despite the crucial role of maternal and child nutrition, few countries have nationally representative dietary intake surveys. To our knowledge, there is a dearth of evidence on the concordance between mother and child dietary diversity and its associated factors in Sub-Saharan Africa (SSA). Therefore, we aimed to examine the magnitude of concordance in mother-child dietary diversity and its associated factors in SSA, using data from the Demographic and Health Surveys (DHS).

## Methods and materials

### Data source

We conducted a community-based cross-sectional study using Demographic and Health Survey (DHS) data from eight sub-Saharan African countries: Burkina Faso (2021), Côte d’Ivoire (2021), Ghana (2022), Kenya (2022), Mozambique (2022/23), Nigeria (2018), Sierra Leone (2023), and Tanzania (2022). These countries provided dietary diversity data for mothers and children aged 6–23 months. The DHS employed a stratified two-stage sampling design, selecting clusters (enumeration areas) in the first stage and systematically sampling households in the second [30]. Our analysis utilized the Individual Record (IR) file, extracting dependent and independent variables based on relevant literature and combining them with the STATA append command. The study included 29,138 weighted samples of reproductive-aged women who had given birth within five years of the survey.

### Outcome variable

The outcome variable, mother-child dietary diversity concordance, was derived from DHS data assessing dietary diversity through 10 questions for mothers and 8 for children. Responses were dichotomized, with “yes” indicating consumption of at least five of ten food groups for mothers and at least four of eight food groups for children within the past 24 hours.

### Operational definition

According to WHO guidelines, minimum dietary diversity is defined as consuming a variety of food groups to meet daily nutrient needs [31]. For children, this entails consuming four or more of eight food groups, including breast milk, grains, roots, legumes, dairy, flesh foods, eggs, and fruits or vegetables rich in vitamin A. For mothers, the threshold is five or more of ten food groups, which include grains, roots, plantains, pulses, nuts, dairy, flesh foods, eggs, dark leafy greens, and other fruits or vegetables.

Dietary concordance refers to the alignment between maternal and child dietary patterns. Concordance occurs when both the mother and child meet their respective dietary diversity thresholds, while discordance reflects a mismatch.

### Independent variables

The analysis accounted for both maternal and child-related factors, such as the mother’s age, media exposure, child’s sex, household wealth, maternal education, marital status, and employment status, all classified as individual-level factors. In contrast, distance to health facilities and the region of sub-Saharan Africa (SSA) were categorized as community-level variables. Media exposure was determined by combining three activities: watching television, reading newspapers, and listening to the radio. Participants were marked as ‘yes’ for media exposure if they engaged in any or all of these activities, and ‘no’ if they did not engage in at least one.

### Data management and analysis

Data were extracted using Stata version 17 software and weighted to ensure representativeness by applying the sampling weight (v005). Due to the hierarchical structure of the DHS data, in which mothers and children are nested within clusters, a mixed-effect binary logistic regression analysis was conducted. Both bivariable and multivariable analyses were performed, with variables that had a P-value of ≤ 0.2 in the bivariable analysis considered as candidates for the multivariable multilevel logistic regression. The Adjusted Odds Ratio (AOR) with a 95% Confidence Interval (CI) was reported to evaluate statistical significance and the strength of the associations. Kappa statistics were employed to assess the extent of agreement between the mother’s and child’s MDD. Additionally, we employed Cohen’s kappa statistic to assess the level of agreement between maternal and child dietary diversity [32].

### Ethical consideration

There was no need for ethical clearance as the researcher did not interact with respondents. The data used was obtained from the MEASURE DHS Program, and permission for data access was obtained from the Measure DHS program through an online request from http://www.dhsprogram.com

## Results

### Descriptive characteristics of study participants

A total of 29,138 women of reproductive age were included in the survey over the five years preceding the study. The majority of study participants were from Nigeria (31.66%) followed by Burkina Faso (11.63%). Approximately 71.25% of the mothers were aged between 20 and 34 years. About 40.06% of the participants had no formal education, and around 22.74% lived in low-income households (Table 1).

**Table 1:** Descriptive characteristics of women aged 15-49 in eight sub-Saharan African countries.

Variables	Weighted frequency	Percentage (%)
**Maternal age (in years)**		
< 20	2373	8.14
20-34	20761	71.25
≥35	6004	20.61
**Maternal education**		
No education	11672	40.06
Primary	7113	24.41
Secondary	8542	29.32
Higher	1810	6.21
**Household wealth status**		
Poorest	6627	22.74
Poorer	6157	21.13
Middle	5820	19.98
Richer	5585	19.17
Richest	4947	16.98
**Media exposure**		
No	10628	36.47
Yes	18510	63.53
**Number of gestations**		
Single	28191	96.75
Multiple	948	3.25
**Sex of child**		
Male	14799	50.79
Female	14339	49.21
**Number of household member**		
≤ 5	4012	13.77
6 – 10	11253	45.48
> 10	11872	40.75
**Distance to health facility**		
A big problem	10236	35.14
Not a big problem	18896	64.86
**Marital status**		
Single	1906	6.54
Married	25937	89.01
Widowed or divorced	1296	4.45
**Country**		
Burkina Faso	3390	11.63
Cote d’Ivoire	2705	9.28
Ghana	2663	9.14
Kenya	2587	8.88
Mozambique	2707	9.29
Nigeria	9225	31.66
Serra Leone	2671	9.17
Tanzania	3191	10.95

### Dietary consumption pattern of mothers and children

Approximately 40% of mothers and 83.15% of children consume staple foods. About one-third (33.64%) of mothers incorporate pulses, such as beans and lentils, into their diets. Approximately 24.53% of both mothers and children reported consuming nuts in the past 24 hours. In terms of meat, poultry, and fish, around 63.29% of mothers and 38.40% of children indicated that they had eaten these foods. Nearly 60% of mothers included dark green leafy vegetables in their diets, while around 39.42% of children consumed fruits and vegetables rich in Vitamin A within the last 24 hours (Table 2). Almost 70% (20,667) of women and 82% (24,466) of children did not meet the minimum dietary diversity requirements. Conversely, approximately 30.72% (9,166) of women and 18.00% (5,369) of the sample consumed at least five different food groups.

**Table 2:** Proportion of food groups consumption of mothers and children in the previous 24 hours in eight sub-Saharan African countries.

Minimum Dietary Diversity for mothers %		Minimum Dietary Diversity for child %	
1. Grains, white roots and tubers, and plantains	39.95%	1. Breast milk	75.94%
2. Pulses (beans, peas, and lentils)	33.64%	2. Grains, roots, and tubers	83.15%
3. Nuts and seeds	24.53%		
4. Dairy	19.87%	3. Legumes and nuts	23.70%
5. Meat, poultry, and fish	63.29%	4. Dairy products	23.32%
6. Eggs	11.14%	5. Flesh foods (meat, fish, poultry, and liver/organ meats)	38.40%
7. Dark green leafy vegetables	59.86%	6. Eggs	11.68%
8. Other vitamin A-rich fruits and vegetables	16.87%	7. Vitamin A-rich fruits and vegetables	39.42%
9. Other vegetables	51.98%	8 Other fruits and vegetables	17.34%
10. Other fruits	23.92%		

### Proportion of mother-child pair dietary diversity concordance

The dietary diversity concordance among mother-child pairs in Sub-Saharan Africa (SSA) was found to be 74.48% (95% CI: 73.98, 74.98). Of these pairs, 62.81% had both the mother and child consuming a limited variety of foods (≤ 5 food groups). In contrast, 11.53% of the pairs exhibited high dietary diversity for both the mother and child (Table 3).

**Table 3:** Minimum dietary diversity concordance between mothers and children aged 6 – 23 months.

		MDD of children		Total
		No	Yes	
**MDD of mothers**	**No**	18739 (62.81%)	1928 (6.46%)	20667 (69.28%)
	**Yes**	5725 (19.19%)	3441 (11.53%)	9166 (30.72%)
**Total**		24464 (82%)	5369 (18%)	29833 (100%)

*MDD- Minimum Dietary Diversity.

### The strength of concordance in mother-child dietary diversity

Cohen’s kappa analysis was conducted to assess the level of agreement between maternal minimum dietary diversity and the dietary diversity of children aged 6 to 23 months. The analysis produced a Cohen’s kappa coefficient of 0.3189 (P < 0.001), indicating fair agreement between mother-child pairs in terms of dietary diversity. The overall agreement between maternal and child dietary diversity was 74.35%, indicating substantial agreement between the mothers’ dietary diversity and that of their children.

### Factors associated with maternal and child dietary diversity concordance

We evaluated whether the mixed-effect binary logistic regression model offered a better fit than the single-level binary logistic regression model by using the Likelihood Ratio (LR) test. The LR test yielded statistically significant results (p < 0.05), indicating that the multilevel model fit better than the single-level model. A bivariable analysis was performed between the outcome variable and several explanatory variables, including maternal education, media exposure, household wealth status, twin pregnancies, the number of household members, and the child’s sex. Variables with a p-value less than 0.2 in the bivariable analysis were considered for inclusion in the multivariable logistic regression model. The final model was adjusted for these selected explanatory variables. The odds of dietary diversity concordance between mother-child pairs were 1.42 times higher (AOR = 1.42, 95% CI: 1.31, 1.53) for mothers with primary education compared to those with no education. In contrast, the odds of concordance in dietary diversity were 25% lower (AOR = 0.75, 95% CI: 0.66, 0.88) for mothers with higher education compared to those with no education. The odds of concordance in dietary diversity for mother-child pairs were 19% lower (AOR = 0.81, 95% CI: 0.74, 0.88) for mothers from poorer households, 18% lower (AOR = 0.82, 95% CI: 0.75, 0.89) for those from middle-income households, 20% lower (AOR = 0.80, 95% CI: 0.73, 0.88) for those from richer households, and 25% lower (AOR = 0.75, 95% CI: 0.67, 0.83) for those from the wealthiest households, compared to mothers from the poorest households. Additionally, the odds of concordance were 22% lower for mothers with media exposure compared to those without it (AOR = 0.78, 95% CI: 0.73, 0.83). The odds of dietary diversity concordance with their children in mothers who were divorced or widowed were 1.54 times (AOR = 1.54, 95% CI: 1.29, 1.84) compared to single mothers (Table 4).

**Table 4:** Multilevel binary logistic regression analysis of factors associated with maternal-child dietary diversity concordance in SSA.

Variables	Dietary diversity concordance		Crude Odds Ratio (COR) with the 95% CI	Adjusted Odds Ratio (AOR) with the 95% CI
	Discordant	Concordant		
Mothers age (in years)				
< 20	592	1874	1	1
20-34	5458	15626	0.91 (0.83, 1.01)	0.97 (0.87, 1.07)
≥ 35	1603	4680	0.93 (0.83, 1.04)	0.94 (0.83, 1.06)
Mothers educational status				
No	3049	9447	1	1
Primary	1431	5596	1.30 (1.21, 1.40)	1.42 (1.31, 1.53)
Secondary	2560	6107	0.79 (0.74, 0.84)	0.96 (0.89, 1.03)
Higher	613	1030	0.57 (0.51, 0.63)	0.75 (0.66, 0.85)
Household wealth status				
Poorest	1544	5896	1	1
Poorer	1611	4823	0.78 (0.72, 0.85)	0.81 (0.74, 0.88)
Middle	1587	4564	0.75 (0.70, 0.82)	0.82 (0.75, 0.89)
Richer	1528	3967	0.69 (0.64, 0.75)	0.80 (0.73, 0.88)
Richest	1383	2930	0.57 (0.52, 0.62)	0.75 (0.67, 0.83)
Media exposure				
No	2411	8950	1	1
Yes	5242	13230	0.70 (0.66, 0.74)	0.78 (0.73, 0.83)
Sex of child				
Male	3937	11226	1	1
Female	3716	10954	1.03 (0.98, 1.09)	0.96 (0.89, 1.04)
Family size				
≤ 5	1037	2805	1	1
6 – 10	3556	9685	1.01 (0.93, 1.10)	0.98 (0.90, 1.06)
> 10	3060	9690	1.16 (1.06, 1.26)	1.08 (0.99, 1.18)
Number of gestations				
Single	7384	21452	1	1
Multiple	269	728	0.94 (0.81, 1.08)	0.93 (0.81, 1.08)
Distance to health facility				
A big problem	2619	8217	1	1
Not a big problem	5034	13961	0.90 (0.85, 0.95)	1.02 (0.97, 1.09)
Marital status				
Single	549	1360	1	1
Married	6850	19764	1.15 (1.03, 1.28)	1.11 (0.99, 1.24)
Divorced or widowed	254	1056	1.69 (1.42, 2.01)	1.54 (1.29, 1.84)

***Abbreviation:*** *CI = Confidence Interval*

## Discussion

We found a strong relationship between mothers and children’s dietary diversity in SSA (Kappa = 0.3189, p < 0.001, and an overall level of agreement of 74.35%). It was supported by studies reported in developing countries [24,33], the findings may be attributed to the implementation of health extension programs and the formation of health development armies in several African countries. These initiatives are designed to educate communities about various health interventions, including maternal and child nutrition practices. The results suggest a well-documented association between mothers with access to diverse diets and their children achieving minimum dietary diversity, and vice versa. This strong correlation between mothers’ and children’s dietary diversity may explain the high level of agreement observed between the two groups’ MDD.

About three-fourths of mother-child pairs had concordant dietary diversity patterns in SSA. It was lower than a previous study [34]. In sub-Saharan African countries, deep-rooted poverty often limits families’ ability to afford a diverse diet for both mothers and children [35]. As a result, mothers frequently prioritize their children’s nutrition, which can lead to significant differences in dietary diversity patterns between mothers and their children [36]. Moreover, variations in the magnitude of minimum dietary diversity concordance between mothers and children may be influenced by the different number of food groups used to calculate dietary diversity for each group. Additionally, the economic conditions of these countries can exacerbate these disparities, as limited access to a variety of foods further widens the gap in dietary diversity between mothers and their children.

Maternal educational status, household wealth status, marital status, and media exposure were significantly associated with concordance in dietary diversity between mothers and their children. Mothers with primary education were more likely to exhibit dietary diversity concordance with their children compared to mothers without formal education. However, mothers with higher education levels had lower odds of achieving concordant dietary diversity with their children compared to those without formal education. It was supported by previous studies [33,34,37], the observed association may be explained by the fact that mothers with higher levels of education could be more engaged in various activities, leaving them with less time for optimal childcare [38]. Tailored interventions aimed at this group may help address micronutrient deficiencies in children. In contrast, mothers with a primary level of education tend to show higher trust in information and greater awareness of dietary practices compared to women without formal education [39].

Mothers belonging to the poorer, middle, richer, and richest households had lower odds of dietary diversity concordance with their children compared to mothers belonging to the poorest households. It is consistent with studies reported in Nigeria [21] and Senegal [40]. In poor households, there is often less access to a variety of foods, leading to simpler and more homogeneous diets for both mothers and children [41]. As a result, their diets tend to align more closely. Additionally, in low-income households, mothers may be more directly involved in meal preparation and food selection, creating a stronger link between their dietary habits and those of their children. In wealthier households, there is generally greater access to diverse foods, and dietary patterns may differ between mothers and their children due to factors like varied food preferences, higher consumption of processed or convenience foods, and potentially less direct involvement of mothers in meal preparation [42]. This can lead to a mismatch in the dietary diversity between mothers and their children, as children may be more exposed to different food environments, such as eating at school or with caregivers, where their diet may differ from their mothers.

Mothers who had media exposure had lower odds of dietary diversity concordance with their children. It was in line with previous studies [16,43], it could be because media exposure can introduce mothers to a variety of nutritional information, but not all of it may be accurate or contextually relevant [44]. Some media content could promote unhealthy eating habits, focus on convenience foods, or emphasize modern diets that deviate from traditional, more diverse dietary practices. Additionally, exposure to media might lead to time constraints, as mothers may spend more time engaging with media, reducing the time spent preparing balanced and diverse meals for their children. This could ultimately result in a disconnect between what the mother consumes and what she provides for her children, leading to lower dietary diversity concordance.

Another significant predictor of dietary diversity concordance among mothers-child pairs was marital status. Being divorced or widowed increased the odds of having dietary diversity concordance with their children compared to single women. It was consistent with studies reported in Ghana [24] and SSA [45], it could be due to divorced or widowed women having higher odds of aligning their dietary diversity with their children’s compared to single women, potentially due to increased responsibilities or prioritization of family health after the loss of a partner, which may influence their food choices and household meal planning.

The findings of this study should be interpreted in light of the following strengths and weaknesses. First, it was based on a nationally representative sample of eight Sub-Saharan African (SSA) countries to investigate factors associated with dietary diversity concordance among mothers and children aged 6–23 months, allowing the results to be generalized to similar populations in SSA. Second, except a few food groups, the same food groups were assessed for both mothers and their children, enabling a direct comparison of maternal and child dietary diversity.

On the other hand, this study utilized a cross-sectional DHS survey, which limits our ability to infer causal relationships between factors and dietary diversity concordance. Additionally, dietary data was collected using a 24-hour recall, which introduces the possibility of recall bias and social desirability bias. A further limitation is the reliance on the 24-hour recall method, which may affect the accuracy of the reported food intake. Moreover, we acknowledge the limitation that our study relies solely on quantitative data from the DHS and lacks qualitative insights into cultural, religious, and social factors influencing dietary diversity.

## Conclusions

This study found a moderate concordance between child and maternal dietary diversity in Sub-Saharan Africa (SSA), with approximately three-fourths of the dietary diversity between mothers and children being concordant. Factors such as maternal education, household wealth, marital status, and media exposure were significantly associated with dietary diversity concordance. These findings highlight the need for targeted policy interventions aimed at promoting community-based education on the importance of dietary diversity for both mothers and children, particularly for women from economically advantaged households. Policies should focus on improving access to diverse and nutritious foods, especially in low-income and rural areas. Furthermore, there is a pressing need to enhance the quality and relevance of information on dietary diversity, ensuring that it is culturally appropriate, accessible, and effectively communicated to mothers across different socio-economic backgrounds. By addressing these factors, policies can help reduce disparities in dietary diversity and improve overall health outcomes for mothers and children in SSA.

## Abbreviations

AOR,: Adjusted Odds Ratio
CI: Confidence Interval
DD: Dietary diversity
DHS: Demographic and Health Survey
EAs: Enumeration Areas
IR: Individual Record
LLR: Log Likelihood Ratio
LMICs: Low- and middle-income countries
MDD: Minimum Dietary Diversity
SSA: sub-Saharan Africa
WHO: World Health Organization
